# Sequential ATR and PARP inhibition overcomes acquired DNA damaging agent resistance in pancreatic ductal adenocarcinoma

**DOI:** 10.1038/s41416-025-03051-z

**Published:** 2025-05-29

**Authors:** Katharine J. Herbert, Rosie Upstill-Goddard, Stephan B. Dreyer, Selma Rebus, Christian Pilarsky, Mukhopadhyay Debabrata, Christopher J. Lord, Andrew V. Biankin, Fieke E. M. Froeling, David K. Chang

**Affiliations:** 1 Wolfson Wohl Cancer Research Centre, School of Cancer Sciences, University of Glasgow, Glasgow, G61 1QH UK; 2 West of Scotland Pancreatic Unit, Glasgow Royal Infirmary, Glasgow, G4 0SF UK; 3 Department of Surgery, Universitätsklinikum Erlangen, Friedrich-Alexander-Universität Erlangen-Nürnberg (FAU), 91054 Erlangen, Germany; 4 Department of Biochemistry and Molecular Biology, Mayo Clinic College of Medicine and Science, Jacksonville, FL 32224 USA; 5 CRUK Gene Function Laboratory and Breast Cancer Now Toby Robins Research Centre, The Institute of Cancer Research, London, SW3 6JB UK; 6 Department of Oncology, Beatson West of Scotland Cancer Centre, Glasgow, G12 0YN UK

**Keywords:** Cancer models, Cancer therapeutic resistance

## Abstract

**Background:**

Pancreatic ductal adenocarcinoma (PDAC) remains the most lethal cancer. While DNA damaging agents such as platinum and PARP inhibitors have derived clinical benefits, acquired resistance invariably develops. Hence there is an urgent need for novel therapeutic strategies to overcome acquired resistance.

**Methods:**

Clinically relevant resistance in PDAC patient-derived cell lines was achieved by extended exposure to chemotherapy agents. Synergy scoring, clonogenicity, flow cytometry, immunofluorescence, and transcriptomic analysis were used to investigate the efficacy of ATR (ceralasertib) and PARP (olaparib) inhibitors in overcoming acquired resistance.

**Results:**

Acquired resistance was associated with transcriptomic shifts in cell cycle checkpoint regulation, metabolic control, DNA damage response (DDR), programmed cell death, and the replication stress response. Combination treatment with ceralasertib, and olaparib was synergistic in all models of acquired resistance. Sequential use of ceralasertib prior to olaparib was highly effective at low dose for DDR proficient models, whereas DDR deficient models responded better with olaparib treatment first.

**Conclusions:**

We provide in vitro evidence of a novel therapeutic strategy to overcome acquired resistance to PARP inhibitor and platinum in PDAC, using sequential exposure to ceralasertib and olaparib. A sequential regimen should be investigated clinically to circumvent dose limiting toxicity seen in concurrent combinations.

## Introduction

Outcomes for pancreatic ductal adenocarcinomas (PDAC) have not changed significantly over the last 50 years with an overall 5-year survival of ~8% [1], and is predicted to be the second leading cause of cancer related death by 2030 [2]. Reasons for the poor prognosis are multifactorial. Most of the patients present with advanced disease as symptoms are generally non-specific, and many of the patients have rapidly deteriorating performance status, unable to receive any active treatments [3]. In addition, overall response rate for standard chemotherapy is modest and tumours eventually acquire resistance even after a period of treatment response.

Tumour molecular profiling through next generation sequencing has been the foundation of modern oncology to identify targeted therapy that the patient is most likely going to respond to [4, 5]. One of the most promising actionable segments are those whose tumours with homologous recombination deficiency (HRD) [6], which preferentially respond to DNA damaging agents such as platinum and poly (ADP-ribose) polymerase (PARP) inhibitors through synthetic lethality. This approach has significantly improved the outcomes of breast (OlympiAD, OlympiA), prostate (PROfound, PROpel), and ovarian (SOLO-1, PAOLA-1, SOLO-2, and ARIEL3) cancer patients clinically [7–14].

In PDAC, platinum-based chemotherapy such as FOLFIRINOX has demonstrated clinical efficacy in metastatic [15] and adjuvant [16] settings. More recently, maintenance olaparib and rucaparib demonstrated significant progression free survival benefit over placebo in germline *BRCA1/2* carriers and germline and somatic *BRCA1/2*, *PALB2* PDAC respectively after a period of platinum stabilisation [17–19]. However prolonged exposure to platinum and PARP inhibitors eventually leads to treatment failure over time due to acquired resistance. When acquired resistance happens, unfortunately the prognosis is extremely poor as reported by the RUCAPANC authors [20], highlighting an urgent unmet clinical need.

Aberrant coordination of events occurring during replication causing replication stress (RS) is a common feature of cancer due to the close relationship of cell cycle and DNA maintenance machinery [21]. An efficient response to and recovery from replication-induced DNA damage is essential to the maintenance of genome wide fidelity, with most cancers developing a degree of enhanced innate tolerance to RS [22] which in turn is associated with chemotherapy resistance. This may create a dependency on the ataxia telangiectasia mutated and rad3 related kinase (ATR) /CHEK1/WEE1 signalling axis, that coordinates the replication stress response (RSR), and represents an exploitable vulnerability in treatment resistant cancers [23, 24]. We previously reported a RS signature score based on molecular processes involved in maintenance of genomic integrity and demonstrated its correlation to cell cycle checkpoint inhibitor responsiveness such as ATR and CHK in preclinical models of PDAC [25]. Clinically, ATR inhibitors have demonstrated efficacy by differentially targeting tumours with high RS [26]. In addition, early trial results of ATR inhibitor BAY1895344 have shown clinical efficacy in a wide range of heavily pre-treated solid cancers including those acquired resistance to PARP inhibitors [27]. The authors also found evidence of increased DNA damage in on-treatment biopsies, which is unsurprising as acquired PARP inhibitor resistance may be mediated by ATR-induced protection of the replication fork [28]. This further supports the combination of ATR inhibitor to DNA damaging agents as a viable strategy to overcome acquired resistance to DNA damaging agents, even though early phase ATR inhibitor trials showed that intermittent on-off dose scheduling is required to improve tolerability [29].

Here, we build on our previous work which investigated combination of ATR inhibitor and DNA damaging agents as novel therapeutic strategy to overcome acquired resistance to DNA damaging agents in PDAC, asking whether scheduling ATR inhibition could effectively improve response and circumvent clinical adverse effects.

## Methods

### Chemicals

Chemotherapy agents - cisplatin (Accord Healthcare, London, UK), rucaparib camsylate (Clovis Oncology, Boulder, CO), olaparib/AZD2281 and ceralasertib/AZD6738 (AstraZeneca, Cambridge, UK). Replication stress inducers – hydroxyurea (HU, Sigma-Aldrich), nocodazole (Cayman Chemical, Ann Arbor, MI). All compounds were prepared as stock solutions in DMSO and stored at −80 °C until needed. Treatments were serially diluted in PBS immediately before addition to culture media.

### Cell culture

Patient derived cell lines were maintained in high (20%) or low (5%) oxygen conditions at 37 °C and 5% CO_2_ as previously described [30–32]. *BRCA2* revertant Capan1 cell lines (described in [33]) was cultured in IMDM with 10% Foetal Bovine Serum (Invitrogen). Cell lines were tested routinely for mycoplasma contamination using MycoAlert PLUS Mycoplasma Detection Kit (Lonza).

### Generation of treatment acquired resistant PDAC cell lines

A *BRCA1* mutated (Ch17: 41,201,216 (c. A > G)) patient-derived cell line [TKCC_10] was sub-cultured in media containing cisplatin, olaparib or rucaparib over a period of 9 months. Concentration of treatment-containing media was increased until resistant clones were able to survive growth in media containing the 5X the IC_50_ (cisplatin) or 10X the IC_50_ (olaparib or rucaparib) compared to the parent cell line (Supplementary Fig. S2a). Where possible, the parent cell line was passage-matched to each resistant clone. Prior to experimental analysis, resistant clones were cultured in fresh treatment-free media for 7–10 days to ensure that results reflected stable change. The *BRCA2* revertant Capan1 cell line (Capan1^BRCA2Revertant^) has been previously described [33].

### RNASeq expression analysis

RNA extractions were performed using QIAGEN RNeasy Mini kit (Cat#74104) according to manufacturer’s specifications. RNAseq libraries were generated by the Beatson Molecular Technology Unit and sequenced by the Glasgow Precision Oncology Laboratory as described previously [25]. Sequencing quality was assessed with FastQC (v 0.11.9) [34] and files were processed with fastp (v 0.21.0) [35] using default settings. Quantification was performed against GRCh38 using Salmon (v 1.4.0) [36]. Salmon quantification results were imported into a DESeqDataSet using DESeq2 (v 1.38.3) [37]. Transcripts were mapped to genes using EnsDb.Hsapiens.v86 (v 2.99.0) [38]. Read count data were filtered to retain only those with normalised counts >= 5 in at least 3 samples (17,403 and 17,080 genes remained in Capan1 samples and TKCC10 samples respectively) and transformed using the DESeq2 ‘vst’ function. All downstream analysis was performed independently for Capan1 cells and TKCC10 cells. Differential expression analysis was performed for the following comparisons: (i) Capan1 parental vs. Capan1^BRCA2Revertant^, (ii) TKCC10 parental vs. TKCC10 cisplatin, (iii) TKCC10 parental vs. TKCC10 olaparib, (iv) TKCC10 parental vs. TKCC10 rucaparib. Volcano plots showing significantly differentially expressed (DE) genes (adjusted *p*-value < 0.05 & absolute(log2FC) > 1) were produced for each comparison with EnhancedVolcano (v 1.16.0) [39]. Heatmaps of DE genes were produced with ComplexHeatmap (v 2.14.0) [40] and circlize (v 0.4.15) [41].

### Transcriptome analysis

Expression-based clustering analysis and heatmaps were generated using Heatmapper (http://www.heatmapper.ca/expression/) [42]. Replication stress scores were calculated for each sample using a previously defined signature of replication stress [25]. Scores were calculated for each gene-set within the replication stress signature with GSVA [43] and individual scores were summed to obtain an overall replication stress score (RS). Gene Set Enrichment Analysis (GSEA) was performed using Hallmark Gene Ontology (GO) genesets from the Molecular Signatures Database (MSigDB) [44] with GSEA software version 4.3.2 [45].

### In vitro viability assays

Cells were seeded in 96 well plates and treated after an overnight settle. For sequential treatment experiments, the initial treatment was removed after 24 h, cells were washed once with PBS, and media containing the second treatment was added to the appropriate wells. Viability was measured 72 h or 8 days later (depending on the compound) using the CellTiter 96 Aqueous non-radioactive cell proliferation assay (Promega, Madison, WI) with a Tecan Infinite200 Pro plate reader (Tecan Trading AG, Männedorf, Germany). Actinomycin D (Sigma-Aldrich, St Louis, MO), drug vehicle (dimethyl sulfoxide [DMSO]), and media-only controls were performed on each individual plate.

### Colony formation assays

Cells were plated as single cell suspensions (500–10,000 cells/well) and left to recover overnight. Treatments were added from concentrated stock solutions directly to seeding media within each well to avoid disturbing the newly attached cells. For sequential treatment, the initial treatment was removed after 24 h, wells gently washed with PBS, and the final treatment added to the appropriate well. In the case of experiments using cisplatin, cells were exposed for 24 h, after which treatment containing media was removed and replaced with fresh media. Colonies were grown for 14–21 days, stained with crystal violet and counted using an automated GelCount plate scanner (Oxford Optronix, Banbury, UK). The plating efficiency (PE) [PE = Average Colony Number/Cells plated] and the surviving fraction (SF) was calculated using [SF = PE_Treated_ /PE_Untreated_]. The significant difference between nonlinear modelled curves was calculated by two-way ANOVA, with survival as the dependent variable and treatment conditions as the independent variables.

### Immunoblotting

Cell lysates were prepared in RIPA lysis buffer (Thermo Scientific) with protease inhibitors (Roche) and phosphatase inhibitors (Sigma) and protein concentration was determined using the BCA assay (Thermo Scientific). Proteins were separated by SDS-PAGE and transferred to PVDF membranes, which were probed by overnight incubation at 4 °C in primary antibody solution. Targets were detected via HRP-conjugated secondary antibodies exposed to chemiluminescence reagent (Millipore) and imaged using a Licor Odyssey XF Imager (Li-Cor, Lincoln, Nebraska). Antibodies used were anti-SLFN11 (ab121731, Abcam); anti-ATR (ab10312, Abcam); anti-Rad51C (NB100-177, Novus Biologicals); anti-Cyclin E (sc-247, Santa Cruz); anti-Rad51 (ab133534, Abcam); anti-pRPA(S4/S8) (ab243866, Abcam); anti-Actin (CST#3700, Cell Signalling Technology); HRP linked anti-rabbit and anti-mouse IgG (CST#7074 & 7076, Cell Signalling Technology). Images were processed with Image Studio Analysis software v 4.0 (Li-Cor, Lincoln, Nebraska).

### Replication fork stall recovery

Cells were treated with hydroxyurea overnight to induce fork stalling. Treatment media was removed, cells were washed twice in prewarmed PBS and released into media containing 100 ng/mL nocodazole to prevent re-entry into G1. Samples were obtained every 2 h for 10 h following release and processed immediately for cytometric analysis. For each HU-treated sample, a non-HU-treated control sample was prepared identically to standardise for potential interference by nocodazole.

### Cell cycle analysis

Samples were labelled by incubating cells with BrdU (Sigma) at 20 μM in growth medium for 30 min and fixed in ice-old 70% ethanol at 4 °C. S phase immunostaining was performed after pepsin/acid digestion using anti-BrdU (1:100, Beckton Dickson) primary antibody, and AlexaFluor488 conjugated secondary antibody (1:500, Molecular Probes). Following incubation, stained cells were pelleted and incubated in a solution containing propidium iodide (Sigma Aldrich) stain (20 μg/mL) and RNase A (200 μg/mL) for 30 min. Samples were scanned using an Attune NxT cytometer (Invitrogen) at a flow rate of 150–300 events/s.

### Foci counting

Cells were grown on coverslips at sub confluent densities and treatments initiated during logarithmic growth phases. At the relevant time points, nonchromatin bound nuclear protein was removed by preincubation of coverslips with CSK extraction buffer (100 mM NaCl, 300 mM Sucrose, 3 mM MgCl2, 10 mM PIPES (pH 7.0), 1 mM EGTA, 0.5% Triton X100) for 10 min on ice, after which samples were fixed with 4% paraformaldehyde for 15 min. Coverslips were blocked with 2% goat serum before overnight incubation with primary antibody at 4 °C [anti-Rad51 (1:500, Santa Cruz), anti-53BP1 (1:1000, Cell Signalling Technology), anti-phospho-RPA2(Ser4/8)(1:5000, Abcam), and anti-phospho-Histone H2A.X (Ser139) (1:2000, Millipore)], followed by the corresponding fluorescent secondary antibodies [AlexaFluor555 (1:1,000, Molecular Probes) or AlexaFluor488 (1:1,000, Molecular Probes)] for 1 h at room temperature. Nuclei were counterstained with 0.5 μg/ml 4′,6-diamidino-2-phenylindole (DAPI; Sigma-Aldrich), and coverslips were mounted using Vectashield (Vector Laboratories). Z stack images were randomly acquired under identical parameters with a Zeiss LSM780 confocal microscope (Zeiss) using a ×63 oil immersion objective. Data are represented as mean ± SEM of two independent experiments evaluating at least 200 nuclei in each experiment.

### Statistical analysis

Zero-interaction potency (ZIP) synergy scoring was calculated using SynergyFinder 3.0 [46]. For 53BP1, pRPA2, γH2AX and RAD51 foci evaluation, images were analysed using FIJI (v2.9.0) [47]. Statistical analysis and graphs were produced using GraphPad Prism v9.4.1 (GraphPad Software, San Diego CA). Foci counting experiments were performed twice, with >4 randomly assigned fields captured by confocal microscopy per condition. For colony formation assays, data are representative of three independent experiments from triplicate wells. Data were analysed by two-way ANOVA, with Bonferroni, Tukey’s, or Sidak’s post-tests used for multiple comparisons. Spearman’s rank correlation tests were used to assess homoscedasticity, and checks were performed for Gaussian distribution during analysis. Differences were considered significant at a *P* value of <0.05, and unless stated, all results are presented as mean ± SD or mean ± SEM as indicated.

## Results

### Higher expression of RS response genes is associated with poor survival in PDAC

**Fig. 1:**
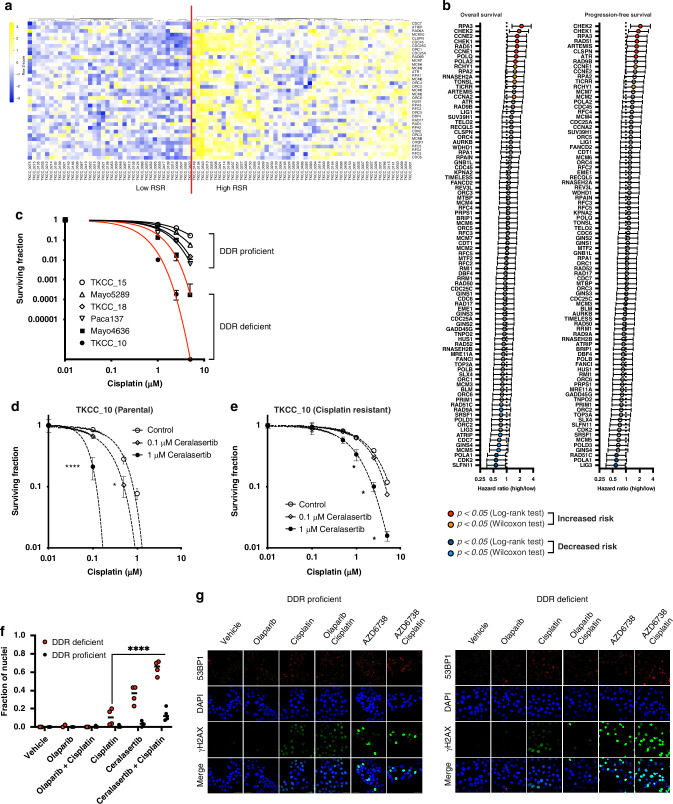
RS and DDR vulnerabilities are prognostic in pancreatic ductal adenocarcinoma. **a** Gene expression and survival data in primary pancreatic ductal adenocarcinomas (PDAC) from ICGC data sets were interrogated to uncover prognostic relationships between replication stress response (RSR) gene expression and DNA damage response (DDR) proficiency. Manhattan clustering of transcriptomic data was used to classify tumours as having high or low RSR potential. **b** Log-Rank testing of survival data was performed to calculate overall and disease-free survival hazard ratios for RSR genes. Survival ratio (± 95% CI) for higher relative expression is shown; genes with lower risk are indicated by blue symbols, and those having significantly increased risk are in red. **c** Clonogenicity assays were performed on cisplatin treated PDCLs, and surviving fraction plotted relative to the plating efficiency of the untreated sample. Curves from DNA damage proficient and DNA resistant cell lines are indicated. Ceralasertib and cisplatin treatment combinations were tested on cisplatin sensitive **d** and cisplatin resistant **e** TKCC10 patient derived cell lines using colony formation assays. Nonlinear regression analysis was performed to determine the effect of overnight treatment for each condition, and individual datasets analysed by 2-way ANOVA with Tukey’s test for multiple comparisons (*, *p* < 0.05; ***, *p* < 0.001). **f,** **g** DDR deficient (TKCC10) and DDR proficient (TKCC26) were exposed to 1 μM concentrations of olaparib, cisplatin, or ceralasertib for 24 hr, probed for γH2AX and imaged by confocal microscopy. Fraction of pan-nuclear γH2AX stained nuclei were analysed by 2-way ANOVA with Tukey’s test for multiple comparisons (****, *p* < 0.0001). Graph is representative of 2 independent experiments, with quantification performed on 4 confocal images containing approximately 20 nuclei per region of interest.

We and others have previously shown that high RS correlated to poorer prognosis in PDAC patients [25] (Supplementary Fig. S1a). To further investigate the prognostic effects of RSR genes, we used expression of genes from the MSigDB Reactome Pathways R-HSA-176187, R-HSA-5656169, and R-HSA-73894 (activation of ATR activation in response to replication stress, termination of translation synthesis, DNA repair), on the ICGC PACA-AU cohort (Fig. 1a; Supplementary Fig. S1b). Tumours with higher expression of *SLFN11, RAD51C, CDK2, LIG3* and *POLA1* were associated with better prognosis (Fig. 1b; Supplementary Fig. S1c), whereas tumours with increased expression of RSR genes such as *Cyclin E1/2, CHEK1/2, RAD51, ATR, DCLRE1/ARTEMIS* and *RPA3* alone or in combination had worse prognosis, making them ideal novel therapeutic targets.

### Inhibition of replication stress response enhances sensitivity to cisplatin in PDAC

We previously showed that RS score correlates to ATR inhibitor responsiveness [25] (Supplementary Fig. S1d) and concluded that both RS and ATR signalling capacity determines tumour viability. To further explore therapeutic strategies, we treated a panel of patient-derived cell lines (PDCLs) that have been previously comprehensively characterised at the genomic and transcriptomic level (Supplementary Table 1) [25], using cisplatin in combination with an ATR inhibitor, ceralasertib. We demonstrated preferential cisplatin response in DDR deficient models (Fig. 1c). The addition of overnight treatment with ceralasertib further increases sensitivity to cisplatin in clonogenicity assays regardless of DDR status (Figs. 1d, e), and was associated with a significant increase in pan-γH2AX staining, consistent with replication catastrophe due to combination treatment (Figs. 1f, g). Based on these results, we further explored if manipulation of RSR with ATR inhibition can overcome acquired treatment resistance in PDAC.

### Generation and characterisation of acquired resistance preclinical models of PDAC

Development of acquired resistance to platinum and PARP inhibitor treatment is common [48] and is associated with poor prognosis. We modelled acquired cisplatin and PARP inhibitor resistance in PDCLs using prolonged exposure to treatment, or *BRCA2* reversion mutation through CRISPR-mediated reversion of gene function.

#### Prolonged exposure

**Fig. 2:**
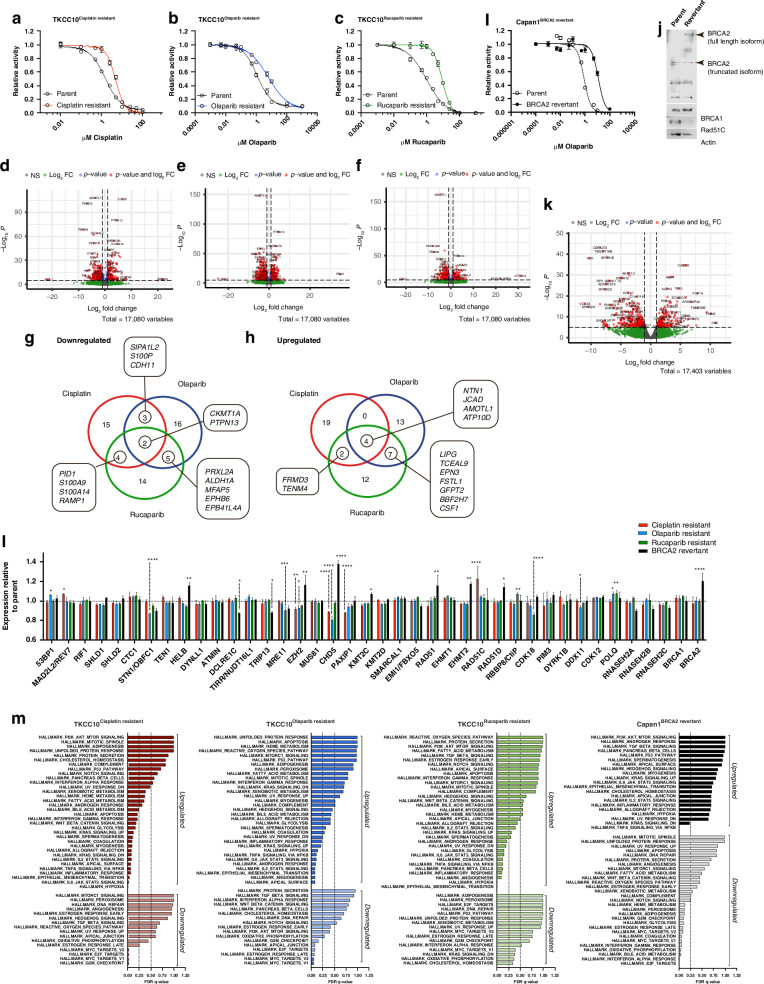
Modelling acquired resistance in PDAC cell lines. **a** Dose-response assay results for cisplatin resistant (Red); **b** Olaparib resistant (Blue); **c** Rucaparib resistant (Green); and **I:** *BRCA2* Revertant (Black). Cell viability was determined using MTS assay and calculated relative to vehicle control. Curves from parent cell lines are indicated by dashed lines (Uncoloured dots). Panels show representative results from 3 independent experiments. Relative activity for cisplatin assay was recorded after 72 h, and after 8 days’ exposure for olaparib and rucaparib. RNASeq was performed on TKCC10 Parental and Treatment Resistant cell lines; and Capan1 Parental and BRCA2 Revertant cell lines (*n* = 3). Volcano plots are of differentially expressed genes from cisplatin resistant (Panel **d**), olaparib resistant (Panel **e**), rucaparib resistant (Panel **f**), and BRCA2 Revertant (Panel **k**) cell lines compared to passage-matched Parental samples. Downregulated (Panel **g**) and upregulated (Panel **h**) differentially expressed genes for TKCC10 resistance cell lines compared to the parental cell line. Panel **J** Immunoblots of Capan1 Parent and BRCA2 Revertant cell lysates probed for BRCA2, BRCA1, RAD51C and Actin protein expression. Multiple BRCA2 isoforms are displayed, arrows indicate truncated and full-length isoforms. Panel **l** Difference in expression of resistance-associated genes relative to parental cell line was analysed by 2-way ANOVA with Dunnett’s test for multiple comparisons (cisplatin resistant, red; olaparib resistant, blue; rucaparib resistant, green; BRCA2 Revertant, black). Results are from 3 independent experiments (*, *p* < 0.05; **, *p* < 0.01; ****, *p* < 0.0001). Panel **m** Enrichment analysis was performed on treatment resistant cell lines using Hallmark gene sets.

We sub cultured TKCC10 (a DDR deficient *BRCA1* mutated PDCL with relatively high basal RS, based on prior analysis [25]) in treatment-spiked growth media (cisplatin, olaparib or rucaparib; 3 independent populations per treatment, cultured in parallel) at increasing concentrations over a 9-month time period to produce resistant colonies which were able to tolerate a five-ten fold higher concentration of the agent compared to the passage matched parental population (Fig. 2a–c; Supplementary Fig. S2a). We then performed RNASeq and analysis for each of the acquired resistant cell lines (Fig. 2d–f; Supplementary Fig. S2b, S2c–S2e). Downregulation of genes implicated in programmed cell death (*CKMT1A*, *PTPN13*) (Fig. 2g), and upregulation of genes promoting cell migration (*NTN1*, *JCAD*, *AMOTL1*, *ATP10D*) (Fig. 2h) are common to all acquired resistant models. While olaparib and rucaparib acquired resistant models generated largely unique transcriptomic profiles, they shared ~ 20% of the differentially expressed genes. These included increased expression of genes regulating multidrug resistance (*TCEAL9*↑), membrane trafficking (*EPN3*↑), cell growth and proliferation (*LIPG*, *BBF2H7*), and promotion of an immune repressive microenvironment (*MFAP5*↓, *CSF1*↑), and downregulation of genes associated with redox homoeostasis (*PRXL2A*↓), and DNA damage response pathway control (*ALDH1A1*↓) (Fig. 2g, h). These data go some way to explaining some of the underlying transcriptional changes driving acquired resistance.

#### Mutation reversion

Reactivation of gene function by reversion mutation is a significant cause of acquired resistance to DNA damaging agents in breast, ovarian and pancreatic cancer [49–51]. Capan1 is a commonly used PDAC cell line harbouring a *BRCA2* frameshift mutation causing truncation of BRCA2 at its DNA binding domain leading to homologous recombination repair deficiency and impedes recovery from replication stress-induced lesions [52]. To recapitulate a reversion mutation seen clinically, we used a Capan1^BRCA2Revertant^ cell line, which harbours a CRISPR-engineered reversion mutation in *BRCA2* that partially restores gene function [33]. This in turn results in a ten-fold lower sensitivity to olaparib (Fig. 2i, j). RNASeq results from the Capan1^BRCA2Revertant^ cell line revealed a significant increase in expression of genes involved in growth and proliferation (*ROBO1*↑), immunosuppression (*HHLA2*↑), and epithelial to mesenchymal transformation (*ADAM8*↑*, FAM3B*↑), and decrease in genes involved in membrane trafficking (*FAM109B*↓), programmed cell death (*CALHM2*↓), cell migration (*IQGAP2*↓) (Fig. 2k; Supplementary S2f) when compared to parental cell line which only carries a dysfunctional *BRCA2* allele.

Previous studies in HR deficient ovarian and breast cancers that have acquired resistance to platinum chemotherapy and/or PARP inhibitor [53, 54] have implicated those genes enriched at collapsed replication forks during ATR inhibition [55]. Our analysis showed changes in 20 DDR and RS associated genes, with 3 affected genes (*EZH2*, *CHD5*, and *POLQ*) common to more than one resistance type. (Fig. 2l).

All acquired resistant cell lines had reduced RS scores compared to parental (Supplementary Fig. S2g), and hallmark pathways analysis revealed downregulation of the G2/M checkpoint, and E2F and MYC targets, and upregulation of hypoxia pathways, promotion of epithelial-mesenchymal transition, and IL2, IL6, TNFα, and KRAS signalling in acquired resistance cell lines (Fig. 2m).

### Acquired resistance is associated with impaired RS tolerance and recovery

**Fig. 3:**
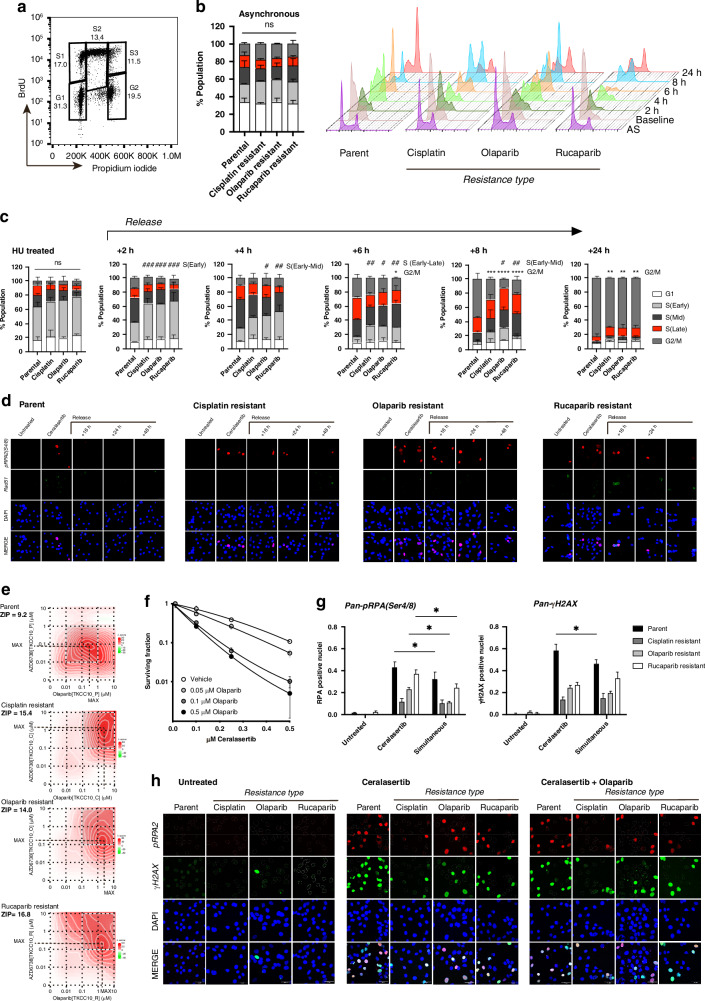
Ceralasertib and Olaparib are synergistic in treatment resistant PDAC. **a** TKCC10 parent and treatment resistant cell lines were synchronised in G1/S by overnight treatment with hydroxyurea and replicating cells were tracked at 2 hourly intervals following release using BrdU uptake. Cell cycle was assayed by propidium iodide staining **b**. Progression of replicating cells through S phase was analysed in 3 phases – Early S(S1), Mid S(S2), and Late S(S3); gating strategy is indicated. Percentage of the total population at each cell cycle phase is shown for each timepoint **c**. TKCC10 parental and resistant cell lines were treated with ceralasertib and olaparib in a 5 × 5 matrix of dose combinations (Ten-fold dilutions, 0–10 μM). Cell viability was assayed by MTS after 8 days’ exposure. Drug synergy was calculated using the interaction potency (ZIP) model across the dose matrix **d**. TKCC10 parent and resistant cell lines were exposed to 1 μM ceralasertib for 5 hr, at which time treatment containing medium was removed and replaced with growth medium. Rad51 and pRPA2 foci were analysed by confocal microscopy at 16hr, 24hr and 48hr following washout. DAPI was used as a nuclear counterstain **e**. Clonogenicity analysis of the effect of ceralasertib and olaparib in combination was performed on the TKCC10 cell line using treatment across a 0–0.5 μM dose range. Surviving fraction was calculated relative to the plating efficiency and analysed by 2-way ANOVA. Survival curves were generated using the linear quadratic model **f**. The response of resistant cell lines to ceralasertib and olaparib combination treatment and ceralasertib monotherapy (1 μM concentrations for each, 24 h exposure) was assayed using Pan-nuclear γH2AX and Pan-nuclear pRPA2 staining as markers for replication catastrophe (Panels **g, h**). Results were analysed by 2-way ANOVA with Tukey’s test for multiple comparisons (*, *p* < 0.05; *ns*, non-significant; results from 2 independent experiments).

We next investigated how acquired resistant PDCLs respond to and recover from exogenous RS induced by treatment with hydroxyurea (HU), a ribonucleotide reductase inhibitor. Cell lines were treated overnight with 1 mM HU to induce replication fork stalling at the G1/S border, and replication progression was tracked by analysing the BrdU positive population in early S (S1), mid-S phase (S2), or late S (S3) following removal of the inhibitor (Fig. 3a). Although we found each acquired resistant cell line had the same percentage of cells in each cell cycle phase as the parental cell line at rest (Fig. 3a), S phase progression is significantly altered in acquired resistance models. Parental TKCC10 cells enter S phase 30 min following release (S1), with most of the population transitioning through mid-S (S2) at approximately 4 h post-release and reaching late S (S3) in 6–8 h. In acquired resistance PDCLs, the transition through S phase is much slower at 8 h post-release, with ~20% of cells failing to fully complete S phase and transition into G2 (Fig. 3b, c) demonstrating impaired tolerance and recover from exogenous RS. Considering the importance of ATR/CHEK1 axis activity in RSR, we treated acquired resistant models with ceralasertib for 72 h and showed decreased sensitivity on viability assay in line with a lower RS score (Supplementary Fig. S3a, b). To test the differences in recovery potential with shorter exposure, we treated these cell lines with 1μM of ceralasertib for 5 h. While we saw the same initial amount of replication-associated damage in the form of pan-nuclear pRPA2 between the parental and the acquired resistant cell lines (Fig. 3d; Supplementary Fig. S3c), the parental cell lines recovered faster after the inhibitor was washed out, with pRPA2 foci retained for at least 48 h in all of the resistant cell lines, and RAD51 foci significantly increased in rucaparib acquired resistance model (Fig. 3d; Supplementary Fig. S3c). These indicate RSR triggered by ATR inhibition is retained following washing out the agent, which opens the possibilities of concurrent or sequential combination with other agents.

### ATR and PARP inhibition are synergistic

Considering the reduction in endogenous RS as shown by reduced RS signature score and reduced sensitivity to ATR inhibition in acquired resistance models, we next asked if manipulation of the RS response by combining ceralasertib and olaparib can re-sensitise and overcome acquired resistance. Olaparib is known to activate the G2/M checkpoint, causing an increase in the G2 population of exposed cells [56]. To assess the effects of olaparib on cell cycle, we treated a panel of PDAC PDCLs with single agent olaparib, and this slowed the production of replication associated damage in acquired resistant cell lines by stalling cell cycle progression at the G2/M border regardless of DDR status (Supplementary Fig. S3d), making it ideal to combine with ATR inhibitor.

Low dose olaparib and ceralasertib treatment used simultaneously over 10–14 days was synergistic for both parental and acquired resistant cell lines (Fig. 3e) even though the parent cell line remained more sensitive than resistant cell lines (MAX Synergy scores: Parent = 9.179; Cisplatin resistant = 15.378; Olaparib resistant = 14.02; Rucaparib resistant = 16.775; Fig. 3e); and significantly reduced clonogenicity for the parent cell line at sub-micromolar concentrations (Fig. 3f). We also observed an increase in the G2 population, albeit for cell lines with high RS scores, where activation of the S phase checkpoint by ceralasertib appears to dominate (Supplementary Fig. S3D). In all but the cisplatin resistant model, overnight exposure to combination treatment reduced replication associated DNA damage (in the form of RAD51 and pRPA foci) when compared to ceralasertib monotherapy (Supplementary Fig. S3c; Supplementary Fig. S4d–g). Using pan-nuclear γH2AX staining as an indicator for replication catastrophe we found that simultaneous combination treatment did not significantly increase replication stress-associated cell death in resistant cell lines when compared to ceralasertib alone (Fig. 3g, h), and produced a small but significant reduction in replication catastrophe measured for the parent cell line.

Considering that sensitivity to olaparib by ceralasertib depends on a combination of S phase exit and replication stress signature, we next investigated if sequentially scheduling treatment can influence sensitivity.

### Scheduled ATR inhibition enhances PARP inhibitor sensitivity in resistant PDAC

**Fig. 4:**
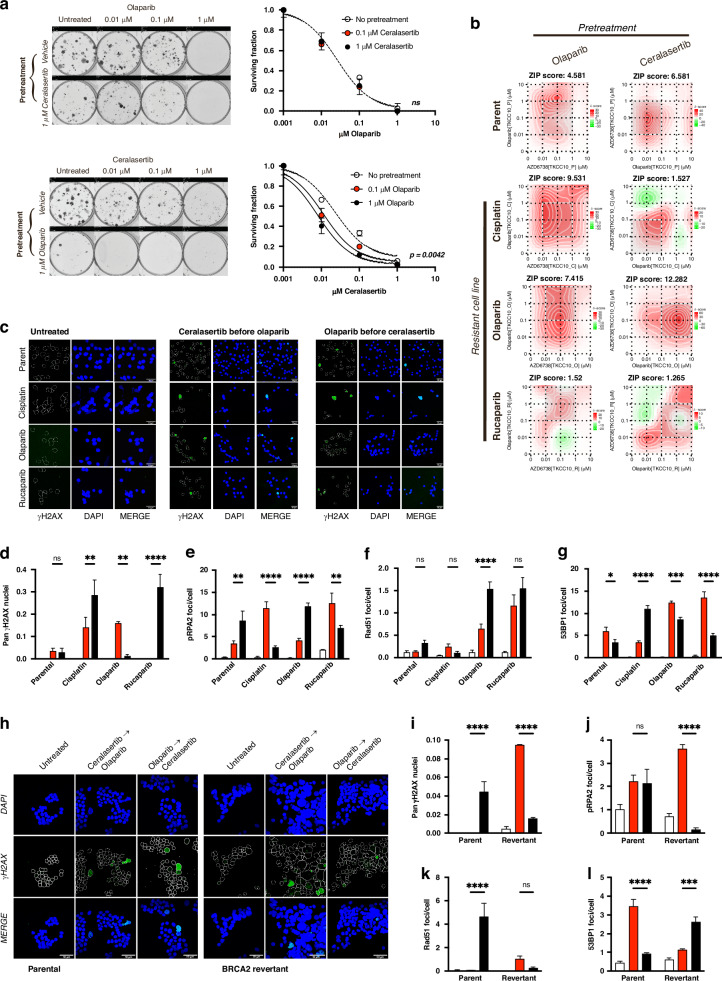
Ceralasertib sensitises acquired resistant PDAC to PARP inhibition. **a** Clonogenicity assay results from the TKCC10 cell line after sequential ceralasertib and olaparib treatment using a matrix of sequential dose combinations. Results were analysed by nonlinear regression relative to the surviving fraction of the vehicle for pretreated (red symbol) or non-pretreated (clear symbol) conditions. Graphs are representative of 2 independent experiments and *p* values for significantly different curves are indicated. Representative clonogenicity assay images are shown. **b** TKCC10 (Parent) and Cisplatin, Olaparib or Rucaparib resistant cell lines were sequentially treated with ceralasertib and olaparib in a 5 × 5 matrix of dose combinations (Ten-fold dilutions, 0–10 μM) in either order. Cell viability was assayed, and drug synergy calculated using the interaction potency (ZIP) model. Dashed lines indicate maximum synergy. TKCC10 acquired resistance cell lines and Capan1 Parental and BRCA2 Revertant cell lines were seeded at sub-confluent densities on coverslips and treated for 24 h with 0.1 μM concentrations of ceralasertib or olaparib prior to 24 h treatment with the alternative agent at 0.1 μM; control (white bars); ceralasertib before olaparib (red bars); olaparib before ceralasertib (black bars). Graphs indicate percentage of cells for each condition with Pan-γH2AX stained nuclei **d**, **i**, pRPA2 foci per cell **e**, **j** Rad51 foci per cell **f**, **k** and 53BP1 foci per cell **g**, **l**. The effect of combination was analysed by 2-way ANOVA with Tukey’s test for multiple comparisons. Conditions were compared to vehicle control (*p* < 0.001, ###; *p* < 0.0001, ####), and effect of alternative treatment order was compared (*, *p* < 0.05; **, *p* < 0.01; ****, *p* < 0.0001). Results are from 2 independent experiments, with at least 4 different fields of view captured for image analysis. Representative confocal images from Pan-γH2AX staining are shown in TKCC10 acquired resistance cell lines **c** and Capan1 Parental and BRCA2 Revertant **h**.

We first assessed clonogenicity on the Parental TKCC10 cell line by treating with one agent followed by treatment with the second agent with each treatment completely washed out with PBS after overnight exposure. We found treatment sequence order significantly influenced the outcome. When ceralasertib was used first, there was no dose-dependent effect on clonogenic synergy, whereas sensitisation increased when cells were treated with olaparib first (Fig. 4a). We then assessed the effect of sequential treatment on acquired resistance models with 24 h exposure to either ceralasertib or olaparib prior to treatment with the alternative agent (using a viability matrix across the same dose range as Fig. 3e).

The efficacy of ceralasertib was enhanced ten-fold by prior exposure to olaparib using the acquired cisplatin resistant model. With the acquired olaparib resistant model, sensitivity to olaparib was enhanced by previous ceralasertib treatment, and with the acquired rucaparib model sensitivity to rucaparib increased with prior exposure to ceralasertib (Fig. 4b; Supplementary Fig. S4a and b). In either case the opposite sequential order did not produce a synergistic response of the same magnitude. These data demonstrate that sensitivity to each PARP inhibiting agent is dependent on adaptation to prior exposure and indicate that the element driving resistance should be used as backbone in combination with ATR inhibition.

We then investigated the underlying mechanisms contributing to different synergy and sensitivity of various drug sequence and regimens in different models of acquired resistance. The percentage of pan-nuclear γH2AX positive cells generated when ceralasertib was used prior to olaparib was significantly greater for the olaparib resistant model, however in cisplatin and rucaparib acquired resistance models, the opposite was true (Fig. 4c, d). This pattern of enhanced DNA damage sensitivity associated with specific treatment order is in line with the response for each resistance model seen in Fig. 4b. To better understand if sensitivity to treatment order is connected to each model’s capacity to recover from replication-associated DNA damage, we next measured the expression of nuclear markers for single strand breaks (pRPA2 foci; Fig. 4e), and double strand breaks (Rad51; Fig. 4f) as well as 53BP1 Foci (Fig. 4g). Expression of these markers are in-line with the treatment sequence and regimens producing peak synergy for each acquired resistance models. For both Olaparib and Rucaparib acquired resistance models, significantly more Rad51 foci were observed with sequential treatment, indicating a greater reliance on these factors for SSB and DSB stabilisation and repair.

We next investigated if the same patterns of treatment sensitivity are seen in models of resistance acquired through reversion mutation mechanism using Capan1 and Capan1^*BRCA2revertant*^ isogenic cell lines. Inhibiting the RSR in the DDR proficient Capan1^*BRCA2revertant*^ cell line with ceralasertib prior to olaparib increased residual DNA damage and restored sensitivity to olaparib in viability assay, produced three-fold higher synergy (Supplementary Fig. S4c), and more replication catastrophe (Fig. 4h, i) than by using olaparib first in the sequence. Foci counting assays confirms these findings, showing increased DDR activity with ceralasertib before olaparib for the Capan1^*BRCA2revertant*^ cell line (Fig. 4j–l). Taken together, this demonstrates that treatment sensitivity can potentially be enhanced by combined manipulation of the DDR and RSR pathways in a sequential manner and is effective regardless of the mechanism driving resistance.

### Sequential ATR and PARP inhibition is effective in PDAC PDCLs

**Fig. 5:**
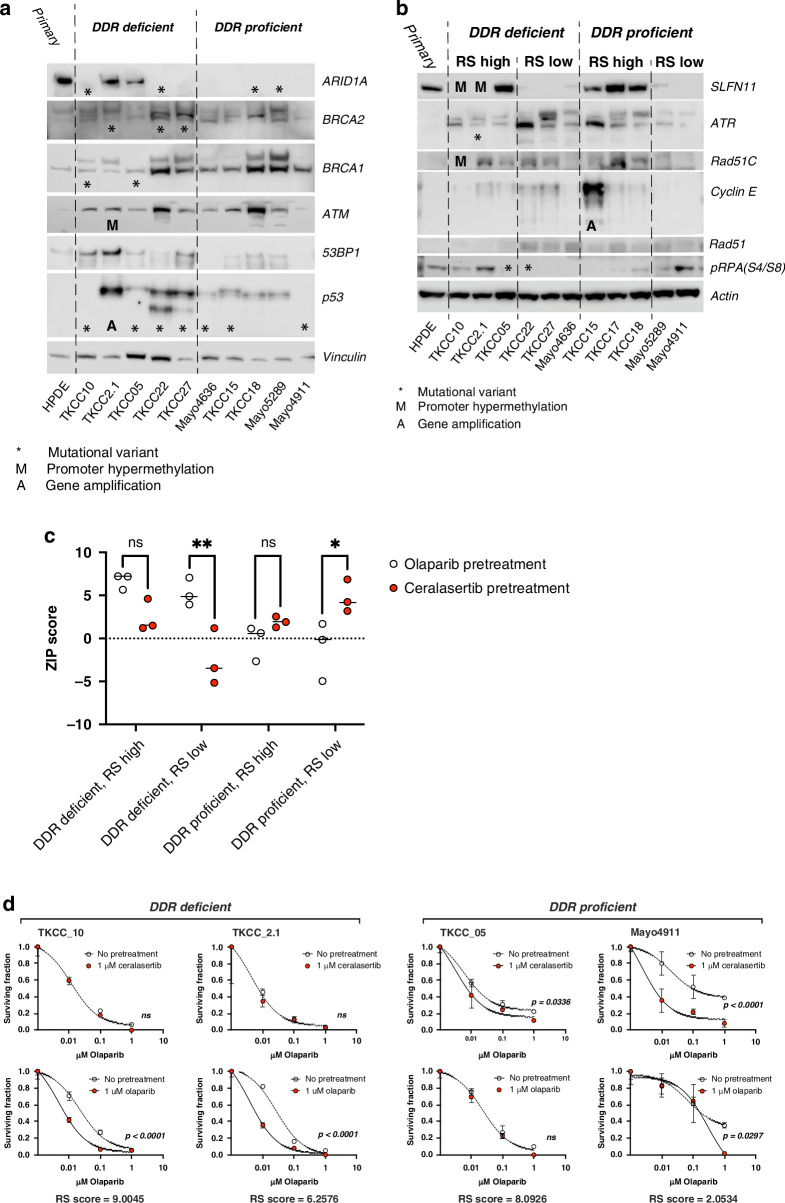
Sequential ATR and PARP inhibitor treatment is effective in PDAC cell lines. **a** Immunoblot from patient-derived cell lines probed for endogenous expression of DNA damage response markers. Cell lines are grouped according to DNA damage response (DDR) proficiency. Mutational variants (*), promoter hypermethylation (M) and amplification (A) of relevant genes for individual cell lines are indicated. **b** Immunoblot from patient-derived cell lines probed for replication stress response markers. Cell lines are grouped according to DNA damage response (DDR) proficiency and replication stress (RS) score (High or Low). Mutational variants (*), promoter hypermethylation (M) and amplification **a** of relevant genes for individual cell lines are indicated. **c** ZIP synergy score analysis for patient-derived cell lines after sequential treatment with ceralasertib and olaparib in a 5 × 5 matrix of dose combinations (Ten-fold dilutions, 0-10 μM). Results are grouped by DNA damage response proficiency and replication stress score (high, H; or low, L), and were analysed by 2-way ANOVA with Sidak’s multiple comparison test (*p < 0.05). **d** Graphical representation of clonogenicity assay results from sequential ceralasertib and olaparib-treated patient-derived cell lines. Results were analysed by nonlinear regression relative to the surviving fraction of the vehicle for pretreated (red symbol) or non-pretreated (clear symbol) conditions. Graphs are representative of 2 independent experiments and *p* values for significantly different curves are indicated.

We next investigated if sequential ceralasertib and olaparib treatment could be effective in the endogenous resistance setting by further screening a panel of 10 PDCLs with varying degree of DDR status and RS signature as previously described [25] for synergy and relative clonogenicity. In these models, *BRCA1* and *BRCA2* loss through mutation are the main cause of DDR deficiency (Fig. 5a), and SLFN11 expression positively correlated to RS signature scores (Fig. 5b, Supplementary Fig. S5a). We found olaparib first then ceralasertib achieved significantly higher synergy scores in DDR deficient / RS high models using viability assays. However, in DDR proficient / RS low models, ceralasertib first then olaparib was more synergistic (Fig. 5c; Supplementary Fig. S5b). Results for clonogenicity mirrored the outcome of synergy scoring assays, with olaparib first sensitising DDR deficient PDCLs to ceralasertib, and ceralasertib first enhancing sensitivity to olaparib for DDR proficient PDCLs (Fig. 5d). However, the role of RS signature is less evident in the different treatment sequence, as cell lines with lower RS scores typically have poor plating efficiency, and colony formation capacity in this assay format. Taken together, the different sequential treatment regimen can potentially be effective in other settings including DDR proficient and a range of RS score models.

## Discussion

DNA damaging agent containing regimens such as FOLFIRINOX and NALIRIFOX [57] as well as PARP inhibitors such as olaparib and rucaparib have demonstrated survival benefits in patients with PDAC, and in some cases exceptional responses that translated to prolonged survival. However, patients who derived meaningful benefits remains the minority, and inevitably, even the responders eventually acquire treatment resistance, which is associated with poor prognosis. Significant efforts have been generated by the scientific and clinical communities to overcome acquired resistance, either with combinatorial regimens, or targeting other aspects of the DNA damage response pathways. Some of the limitations to deploy combinatorial strategy has been dose limiting toxicity as demonstrated by the VIOLETTE clinical trial, where the olaparib plus adavosertib (AZD1775, WEE1 inhibitor) arm was terminated early due to toxicity [58]. The same trial also terminated the concurrent olaparib plus ceralasertib arm due to no additional benefit with the addition of ATR inhibitor over PARP inhibitor alone in acquired platinum resistance triple negative breast cancer [58]. Similarly, while the CAPRI trial using the same olaparib plus ceralasertib regimen as VIOLETTE demonstrated encouraging evidence of clinical benefit in PARP inhibitor acquired resistant high grade serous ovarian cancer [59], there was some tolerability challenges with concurrent administration of the two investigational drugs, likely indicating the requirement of reducing dose intensity compared to respective monotherapies, or intermittent on-off treatments.

Here, we provide in vitro evidence of novel therapeutic strategy of sequential ceralasertib than olaparib to overcome acquired PARP inhibitor and platinum resistance in PDAC models by exploitation of RSR in terms of tolerance and recovery as therapeutic vulnerability. We further demonstrated the sequence of DNA damaging agents and ceralasertib matters as well as the context of the prior line DNA damaging agent exposure. Finally, we showed that we were able to use this sequential treatment regimen of ceralasertib first then olaparib to sensitise a large panel of PDAC models to olaparib regardless of their DNA damage response status and / or innate replication stress level. While current clinical investigations have focused around concurrent doing of Olaparib and ceralsertib with variable dose intensity and on-off regimens, the sequential regimen we have presented here may be a viable alternative strategy to circumvent the challenges of dose limiting toxicity in the combinatorial regimens trailed clinically. The presented novel therapeutic strategy should be further tested in clinically relevant in vivo models of pancreatic cancer before being tested further in well-designed clinical trials for patients who acquired resistance to DNA damaging agents.

## Supplementary information

Supplementary Information

DDR gene variants in PDAC cell lines

Supplementary Figure S1

Supplementary Figure S2

Supplementary Figure S3

Supplementary Figure S4

Supplementary Figure S5

## Data Availability

The data that support the findings of this study are available from the corresponding author upon request.

## References

[1] NHS. *Cancer Survival in England, cancers diagnosed 2016 to 2020, followed up to 2021*, https://digital.nhs.uk/data-and-information/publications/statistical/cancer-survival-in-england/cancers-diagnosed-2016-to-2020-followed-up-to-2021 Accessed 18th May, (2023).

[2] Rahib L, Smith BD, Aizenberg R, Rosenzweig AB, Fleshman JM, Matrisian LM. Projecting cancer incidence and deaths to 2030: the unexpected burden of thyroid, liver, and pancreas cancers in the United States. Cancer Res. 2014;74:2913–21. 24840647 10.1158/0008-5472.CAN-14-0155

[3] Wood LD, Canto MI, Jaffee EM, Simeone DM. Pancreatic cancer: pathogenesis, screening, diagnosis, and treatment. Gastroenterology. 2022;163:386–402.e381. 35398344 10.1053/j.gastro.2022.03.056 PMC9516440

[4] Chan-Seng-Yue M, Kim JC, Wilson GW, Ng K, Figueroa EF, O’Kane GM, et al. Transcription phenotypes of pancreatic cancer are driven by genomic events during tumor evolution. Nat Genet. 2020;52:231–40. 31932696 10.1038/s41588-019-0566-9

[5] Xu Z, Hu K, Bailey P, Springfeld C, Roth S, Kurilov R, et al. Clinical impact of molecular subtyping of pancreatic cancer. Front Cell Dev Biol. 2021;9:743908. 34805152 10.3389/fcell.2021.743908 PMC8603393

[6] O’Kane GM, Lowery MA. Moving the needle on precision medicine in pancreatic cancer. J Clin Oncol. 2022;40:2693–705. 35839440 10.1200/JCO.21.02514

[7] Poveda A, Floquet A, Ledermann JA, Asher R, Penson RT, Oza AM, et al. Final overall survival (OS) results from SOLO2/ENGOT-ov21: A phase III trial assessing maintenance olaparib in patients (pts) with platinum-sensitive, relapsed ovarian cancer and a BRCA mutation. J Clin Oncol. 2020;38:6002.

[8] Robson ME, Tung N, Conte P, Im SA, Senkus E, Xu B, et al. OlympiAD final overall survival and tolerability results: Olaparib versus chemotherapy treatment of physician’s choice in patients with a germline BRCA mutation and HER2-negative metastatic breast cancer. Ann Oncol. 2019;30:558–66. 30689707 10.1093/annonc/mdz012 PMC6503629

[9] Geyer CE Jr, Garber JE, Gelber RD, Yothers G, Taboada M, Ross L, et al. Overall survival in the OlympiA phase III trial of adjuvant olaparib in patients with germline pathogenic variants in BRCA1/2 and high-risk, early breast cancer. Ann Oncol. 2022;33:1250–68. 36228963 10.1016/j.annonc.2022.09.159 PMC10207856

[10] Clarke NW, Armstrong AJ, Thiery-Vuillemin A, Oya M, Shore N, Loredo E, et al. Abiraterone and olaparib for metastatic castration-resistant prostate cancer. NEJM Evid. 2022;1:EVIDoa2200043. 38319800 10.1056/EVIDoa2200043

[11] Mateo J, Bono JSD, Fizazi K, Saad F, Shore N, Sandhu S, et al. Olaparib for the treatment of patients with metastatic castration-resistant prostate cancer and alterations in BRCA1 and/or BRCA2 in the PROfound Trial. J Clin Oncol. 2024;42:571–83. 37963304 10.1200/JCO.23.00339

[12] Ray-Coquard I, Leary A, Pignata S, Cropet C, González-Martín A, Marth C, et al. Olaparib plus bevacizumab first-line maintenance in ovarian cancer: final overall survival results from the PAOLA-1/ENGOT-ov25 trial. Ann Oncol. 2023;34:681–92. 37211045 10.1016/j.annonc.2023.05.005

[13] DiSilvestro P, Banerjee S, Colombo N, Scambia G, Kim B-G, Oaknin A, et al. Overall survival with maintenance Olaparib at a 7-year follow-up in patients with newly diagnosed advanced ovarian cancer and a BRCA mutation: The SOLO1/GOG 3004 Trial. J Clin Oncol. 2023;41:609–17. 36082969 10.1200/JCO.22.01549 PMC9870219

[14] Ledermann JA, Oza AM, Lorusso D, Aghajanian C, Oaknin A, Dean A, et al. Rucaparib for patients with platinum-sensitive, recurrent ovarian carcinoma (ARIEL3): post-progression outcomes and updated safety results from a randomised, placebo-controlled, phase 3 trial. Lancet Oncol. 2020;21:710–22. 32359490 10.1016/S1470-2045(20)30061-9 PMC8210534

[15] Conroy T, Desseigne F, Ychou M, Bouché O, Guimbaud R, Bécouarn Y, et al. FOLFIRINOX versus gemcitabine for metastatic pancreatic cancer. N Engl J Med. 2011;364:1817–25. 21561347 10.1056/NEJMoa1011923

[16] Labori KJ, Bratlie SO, Andersson B, Angelsen J-H, Biörserud C, Björnsson B, et al. Neoadjuvant FOLFIRINOX versus upfront surgery for resectable pancreatic head cancer (NORPACT-1): a multicentre, randomised, phase 2 trial. Lancet Gastroenterol Hepatol. 2024;9:205–17. 38237621 10.1016/S2468-1253(23)00405-3

[17] Golan T, Hammel P, Reni M, Van Cutsem E, Macarulla T, Hall MJ, et al. Maintenance olaparib for germline BRCA-mutated metastatic pancreatic cancer. N Engl J Med. 2019;381:317–27. 31157963 10.1056/NEJMoa1903387 PMC6810605

[18] Reiss KA, Mick R, O’Hara MH, Teitelbaum U, Karasic TB, Schneider C, et al. Phase II study of maintenance rucaparib in patients with platinum-sensitive advanced pancreatic cancer and a pathogenic germline or somatic variant in BRCA1, BRCA2, or PALB2. J Clin Oncol. 2021;39:2497–505. 33970687 10.1200/JCO.21.00003

[19] Tutt ANJ, Garber JE, Kaufman B, Viale G, Fumagalli D, Rastogi P, et al. Adjuvant olaparib for patients with BRCA1- or BRCA2-mutated breast cancer. N Engl J Med. 2021;384:2394–405. 34081848 10.1056/NEJMoa2105215 PMC9126186

[20] Brown TJ, Yablonovitch A, Till JE, Yen J, Kiedrowski LA, Hood R, et al. The clinical implications of reversions in patients with advanced pancreatic cancer and pathogenic variants in BRCA1, BRCA2, or PALB2 after progression on rucaparib. Clin Cancer Res. 2023;29:5207–16. 37486343 10.1158/1078-0432.CCR-23-1467 PMC10806928

[21] Hills SA, Diffley JF. DNA replication and oncogene-induced replicative stress. Curr Biol. 2014;24:R435–444. 24845676 10.1016/j.cub.2014.04.012

[22] Macheret M, Halazonetis TD. DNA replication stress as a hallmark of cancer. Annu Rev Pathol. 2015;10:425–48. 25621662 10.1146/annurev-pathol-012414-040424

[23] da Costa A, Chowdhury D, Shapiro GI, D’Andrea AD, Konstantinopoulos PA. Targeting replication stress in cancer therapy. Nat Rev Drug Discov. 2023;22:38–58. 36202931 10.1038/s41573-022-00558-5 PMC11132912

[24] Ngoi NYL, Pham MM, Tan DSP, Yap TA. Targeting the replication stress response through synthetic lethal strategies in cancer medicine. Trends Cancer. 2021;7:930–57. 34215565 10.1016/j.trecan.2021.06.002 PMC8458263

[25] Dreyer SB, Upstill-Goddard R, Paulus-Hock V, Paris C, Lampraki EM, Dray E, et al. Targeting DNA damage response and replication stress in pancreatic cancer. Gastroenterology. 2021;160:362–377.e313. 33039466 10.1053/j.gastro.2020.09.043 PMC8167930

[26] Barnieh FM, Loadman PM, Falconer RA. Progress towards a clinically-successful ATR inhibitor for cancer therapy. Curr Res Pharm Drug Discov. 2021;2:100017. 10.1016/j.crphar.2021.100017 PMC8663972 34909652

[27] Yap TA, Fontana E, Lee EK, Spigel DR, Højgaard M, Lheureux S, et al. Camonsertib in DNA damage response-deficient advanced solid tumors: phase 1 trial results. Nat Med. 2023;29:1400–11. 37277454 10.1038/s41591-023-02399-0 PMC10287555

[28] Yazinski SA, Comaills V, Buisson R, Genois MM, Nguyen HD, Ho CK, et al. ATR inhibition disrupts rewired homologous recombination and fork protection pathways in PARP inhibitor-resistant BRCA-deficient cancer cells. Genes Dev. 2017;31:318–32. 28242626 10.1101/gad.290957.116 PMC5358727

[29] Dillon MT, Guevara J, Mohammed K, Patin EC, Smith SA, Dean E et al. Durable responses to ATR inhibition with ceralasertib in tumors with genomic defects and high inflammation. J Clin Invest 2024;134:e175369. 10.1172/JCI175369 PMC10786692 37934611

[30] Rückert F, Aust D, Böhme I, Werner K, Brandt A, Diamandis EP, et al. Five primary human pancreatic adenocarcinoma cell lines established by the outgrowth method. J Surg Res. 2012;172:29–39. 21683373 10.1016/j.jss.2011.04.021

[31] Waddell N, Pajic M, Patch AM, Chang DK, Kassahn KS, Bailey P, et al. Whole genomes redefine the mutational landscape of pancreatic cancer. Nature. 2015;518:495–501. 25719666 10.1038/nature14169 PMC4523082

[32] Pal K, Pletnev AA, Dutta SK, Wang E, Zhao R, Baral A, et al. Inhibition of endoglin-GIPC interaction inhibits pancreatic cancer cell growth. Mol Cancer Ther. 2014;13:2264–75. 25125675 10.1158/1535-7163.MCT-14-0291 PMC4229952

[33] Drean A, Williamson CT, Brough R, Brandsma I, Menon M, Konde A, et al. Modeling therapy resistance in BRCA1/2-Mutant cancers. Mol Cancer Ther. 2017;16:2022–34. 28619759 10.1158/1535-7163.MCT-17-0098 PMC6157714

[34] FastQC: a quality control tool for high throughput sequence data. v. v 0.11.9 (2010).

[35] Chen S, Zhou Y, Chen Y, Gu J. fastp: an ultra-fast all-in-one FASTQ preprocessor. Bioinformatics. 2018;34:i884–i890. 30423086 10.1093/bioinformatics/bty560 PMC6129281

[36] Patro R, Duggal G, Love MI, Irizarry RA, Kingsford C. Salmon provides fast and bias-aware quantification of transcript expression. Nat Methods. 2017;14:417–9. 28263959 10.1038/nmeth.4197 PMC5600148

[37] Love MI, Huber W, Anders S. Moderated estimation of fold change and dispersion for RNA-seq data with DESeq2. Genome Biol. 2014;15:550. 25516281 10.1186/s13059-014-0550-8 PMC4302049

[38] 2017. EnsDb.Hsapiens.v86: Ensembl based annotation package. v. R package version 2.99.0

[39] 2023. EnhancedVolcano: Publication-ready volcano plots with enhanced colouring and labeling. v. R package version 118.0

[40] Gu Z. Complex heatmap visualization. iMeta. 2022;1:e43. 38868715 10.1002/imt2.43 PMC10989952

[41] Gu Z, Gu L, Eils R, Schlesner M, Brors B. circlize Implements and enhances circular visualization in. R Bioinf. 2014;30:2811–2. 10.1093/bioinformatics/btu393 24930139

[42] Babicki S, Arndt D, Marcu A, Liang Y, Grant JR, Maciejewski A, et al. Heatmapper: web-enabled heat mapping for all. Nucleic Acids Res. 2016;44:W147–153. 27190236 10.1093/nar/gkw419 PMC4987948

[43] Hanzelmann S, Castelo R, Guinney J. GSVA: gene set variation analysis for microarray and RNA-seq data. BMC Bioinf. 2013;14:7. 10.1186/1471-2105-14-7 PMC3618321 23323831

[44] Liberzon A, Subramanian A, Pinchback R, Thorvaldsdóttir H, Tamayo P, Mesirov JP. Molecular signatures database (MSigDB) 3.0. Bioinformatics. 2011;27:1739–40. 21546393 10.1093/bioinformatics/btr260 PMC3106198

[45] Subramanian A, Tamayo P, Mootha VK, Mukherjee S, Ebert BL, Gillette MA, et al. Gene set enrichment analysis: A knowledge-based approach for interpreting genome-wide expression profiles. Proc Natl Acad Sci. 2005;102:15545–50. 16199517 10.1073/pnas.0506580102 PMC1239896

[46] Ianevski A, Giri AK, Aittokallio T. SynergyFinder 3.0: an interactive analysis and consensus interpretation of multi-drug synergies across multiple samples. Nucleic Acids Res. 2022;50:W739–w743. 35580060 10.1093/nar/gkac382 PMC9252834

[47] Schindelin J, Arganda-Carreras I, Frise E, Kaynig V, Longair M, Pietzsch T, et al. Fiji: an open-source platform for biological-image analysis. Nat Methods. 2012;9:676–82. 22743772 10.1038/nmeth.2019 PMC3855844

[48] Waks AG, Cohen O, Kochupurakkal B, Kim D, Dunn CE, Buendia Buendia J, et al. Reversion and non-reversion mechanisms of resistance to PARP inhibitor or platinum chemotherapy in BRCA1/2-mutant metastatic breast cancer. Ann Oncol. 2020;31:590–8. 32245699 10.1016/j.annonc.2020.02.008 PMC7946408

[49] Tobalina L, Armenia J, Irving E, O’Connor MJ, Forment JV. A meta-analysis of reversion mutations in BRCA genes identifies signatures of DNA end-joining repair mechanisms driving therapy resistance. Ann Oncol. 2021;32:103–12. 33091561 10.1016/j.annonc.2020.10.470

[50] Pishvaian MJ, Biankin AV, Bailey P, Chang DK, Laheru D, Wolfgang CL, et al. BRCA2 secondary mutation-mediated resistance to platinum and PARP inhibitor-based therapy in pancreatic cancer. Br J Cancer. 2017;116:1021–6. 28291774 10.1038/bjc.2017.40 PMC5396101

[51] Harvey-Jones E, Raghunandan M, Robbez-Masson L, Magraner-Pardo L, Alaguthurai T, Yablonovitch A, et al. Longitudinal profiling identifies co-occurring BRCA1/2 reversions, TP53BP1, RIF1 and PAXIP1 mutations in PARP inhibitor-resistant advanced breast cancer. Ann Oncol. 2024;35:364–80. 38244928 10.1016/j.annonc.2024.01.003

[52] Schlacher K, Christ N, Siaud N, Egashira A, Wu H, Jasin M. Double-strand break repair-independent role for BRCA2 in blocking stalled replication fork degradation by MRE11. Cell. 2011;145:529–42. 21565612 10.1016/j.cell.2011.03.041 PMC3261725

[53] Labrie M, Brugge JS, Mills GB, Zervantonakis IK. Therapy resistance: opportunities created by adaptive responses to targeted therapies in cancer. Nat Rev Cancer. 2022;22:323–39. 35264777 10.1038/s41568-022-00454-5 PMC9149051

[54] Jackson LM, Moldovan G-L. Mechanisms of PARP1 inhibitor resistance and their implications for cancer treatment. NAR Cancer. 2022;4:zcac042. 10.1093/narcan/zcac042 PMC9773381 36568963

[55] Sirbu BM, McDonald WH, Dungrawala H, Badu-Nkansah A, Kavanaugh GM, Chen Y, et al. Identification of Proteins at Active, Stalled, and Collapsed Replication Forks Using Isolation of Proteins on Nascent DNA (iPOND) coupled with mass spectrometry*. J Biol Chem. 2013;288:31458–67. 24047897 10.1074/jbc.M113.511337 PMC3814742

[56] Lloyd RL, Wijnhoven PWG, Ramos-Montoya A, Wilson Z, Illuzzi G, Falenta K, et al. Combined PARP and ATR inhibition potentiates genome instability and cell death in ATM-deficient cancer cells. Oncogene. 2020;39:4869–83. 32444694 10.1038/s41388-020-1328-y PMC7299845

[57] Nichetti F, Rota S, Ambrosini P, Pircher C, Gusmaroli E, Droz Dit Busset M, et al. NALIRIFOX, FOLFIRINOX, and gemcitabine with nab-paclitaxel as first-line chemotherapy for metastatic pancreatic cancer: a systematic review and meta-analysis. JAMA Netw Open. 2024;7:e2350756. 38190183 10.1001/jamanetworkopen.2023.50756 PMC10774994

[58] Tutt A, Nowecki Z, Szoszkiewicz R, Im SA, Arkenau HT, Armstrong A, et al. 161O VIOLETTE: Randomised phase II study of olaparib (ola) + ceralasertib (cer) or adavosertib (ada) vs ola alone in patients (pts) with metastatic triple-negative breast cancer (mTNBC). Ann Oncol. 2022;33:S194–S195.

[59] Wethington SL, Shah PD, Martin L, Tanyi JL, Latif N, Morgan M, et al. Combination ATR (ceralasertib) and PARP (olaparib) Inhibitor (CAPRI) Trial in Acquired PARP inhibitor–resistant homologous recombination–deficient ovarian cancer. Clin Cancer Res. 2023;29:2800–7. 37097611 10.1158/1078-0432.CCR-22-2444 PMC11934101

