# Supplementary material for: Sequential ATR and PARP inhibition overcomes acquired DNA damaging agent resistance in pancreatic ductal adenocarcinoma
Source: Br J Cancer. 2025 May 29;133(3):381–93. doi: 10.1038/s41416-025-03051-z (PMC12322129; doi:10.1038/s41416-025-03051-z)
Supplement: Supplementary file 1 — Supplementary Information [file 41416_2025_3051_MOESM1_ESM.docx]

**Supplementary Figure Legends**

Supplementary Table S1.

Mutational variants of DNA damage repair (DDR) genes in patient derived PDAC cell lines grouped according to DDR capacity (from Gastroenterology. 2021 Jan;160(1):362-377.e13. doi: 10.1053/j.gastro.2020.09.043. Epub 2020 Oct 9). Cell lines with significantly impaired DDR gene function are classified as DDR deficient, those with partial impairment are classified as intermediate, and those with no overt impairment to DDR function are classified as DDR proficient.

Supplementary Figure S1.

Heatmaps showing Manhattan clustering of ICGC bulk tumour (Panel A) into high or low expression of gene ontology (GO) pathways involved in the DNA damage response and cell cycle control. Delineation of tumour samples into high and low replication stress (RS) score are shown.

Panel B: Correlation analysis of Overall Survival and Progression Free Survival Hazard Ratio results for replication stress recovery gene expression from the ICGC cohort.

Panel C: A high or low replication stress recovery score was calculated for the ICGC cohort using the combined Z score from genes identified as high or low risk in Figure 1B. Survival analysis was performed for overall survival and progression free survival using the median value as a cutoff. Curves were compared by Logrank Test (p values shown).

IC50 values for 72hr exposure to the ATR inhibitor ceralasertib plotted against the replication stress signature scores using cell lines derived from various PDAC tumours (Panel D). Linear regression line is shown.

Supplementary Figure S2.

Panel A: Methodology for generating treatment resistant and passage-matched parental sub-colonies from the TKCC_10 patient derived PDAC cell line.

Panel B: Principal Component Analysis (PCA) map of RNASeq samples from parental cell line (TKCC10_P), cisplatin resistant cell line (TKCC10_C), olaparib resistant cell line (TKCC10_O), and rucaparib resistant cell line (TKCC10_R). Points refer to independently generated replicate.

Differential gene expression (Top 50) heatmaps for TKCC10 cisplatin resistant (Panel C; TKCC10_C), olaparib resistant (Panel D; TKCC10_O), and rucaparib resistant (Panel E; TKCC10_R) cell lines relative to vehicle treated parental control (TKCC10_P).

Panel F: Differential gene expression (Top 50) heatmap for Capan1 BRCA2 Revertant (Capan1_BRCA2_Rev) relative to the Parental cell line (Capan1_P).

Panel G: Graphical representation of replication stress scores (RS) calculated for TKCC10 parental and resistant cell lines, and Capan1 Parental and BRCA2 Revertant cell lines (Bars indicate mean ±SEM; n = 3).

Supplementary Figure S3.

Panels A & B: Dose-response viability assay curves for 72 hours ceralasertib exposure in TKCC10 acquired resistance (Panel A) and Capan1 BRCA2 Revertant (Panel B) cell lines.

Panel C: TKCC10 parent and treatment resistant cell lines were seeded on coverslips and treated for 5 hours with Vehicle or 1 μM ceralasertib. Treated cells were released from treatments by washing the coverslips twice with PBS. Culture media was replaced and treated coverslips were sampled at 16, 24, and 48 hour timepoints following release. Coverslips were probed for Rad51 foci and pan-nuclear phospho-RPA(S4/S8) expression. Nuclei were counterstained with DAPI. Coverslips were mounted and imaged by confocal microscopy (5 images/condition sampled randomly, representative images shown).

Panel F: Cell cycle cytometric analysis of cell lines with high replication stress score (TKCC02.1); and low replication stress score (TKCC26) after overnight treatment with 1 μM concentrations of olaparib, ceralasertib, or both olaparib and ceralasertib combined. Nuclear DNA content was stained with propidium iodide (PI), and cell cycle populations were analysed using FlowJo software. Images are representative of 2 replicates.

Supplementary Figure S4.

Graphical representation of the zero-interaction potency (ZIP) scores for TKCC10 (Parent) and Cisplatin, Olaparib or Rucaparib resistant cell lines sequentially treated with ceralasertib and olaparib (Panel A); TKCC10 acquired resistance cell lines treated with ceralasertib and rucaparib (Panel B); and Capan1 Parent and BRCA2 Revertant cell lines treated with ceralasertib and olaparib (Panel C).Treatments were administered in either order, with an initial 24 hr pretreatment, followed by 8 days exposure to the Post-treatment in a 5 × 5 matrix of dose combinations (10-fold dilutions, 0-10 μM).

Panels D-G: TKCC10 Parent, Cisplatin, Olaparib or Rucaparib resistant cell lines were treated overnight with 1μM or 10μM concentrations of ceralasertib and/or olaparib and cell lysates screened for relative expression of DNA damage response and replication stress response proteins [γH2AX, RPA32 (phosphorylated at Ser4 and Ser8), CHEK1 (total and phosphoSer345), CHEK2 (total and phosphoThr68) and PARP1 (full length and cleaved)] by immunoblotting. Vinculin expression was used as a protein loading control. Chemiluminescence capture conditions were standardised between blots for each target.

Supplementary Figure S5.

Panel A: Heatmap showing high and low clustering of replication stress (RS) scoring data from ICGC patient derived cell lines using gene sets involved in the DNA damage response and cell cycle control.

Panel B: Combined synergy scores (CSS) for patient-derived cell lines after sequential treatment with ceralasertib. Uncoloured points represent values from cell lines pretreated with olaparib, and red point indicate values from those pretreated with ceralasertib. Results are grouped by DNA damage response proficiency and replication stress score (high, H; or low, L), and were analysed by 2-way ANOVA with Sidak’s multiple comparison test (* p < 0.05; ** p < 0.01; *** p < 0.001).
