# Supplementary figures and images for: Sequential ATR and PARP inhibition overcomes acquired DNA damaging agent resistance in pancreatic ductal adenocarcinoma
Source: Br J Cancer. 2025 May 29;133(3):381–93. doi: 10.1038/s41416-025-03051-z (PMC12322129; doi:10.1038/s41416-025-03051-z)

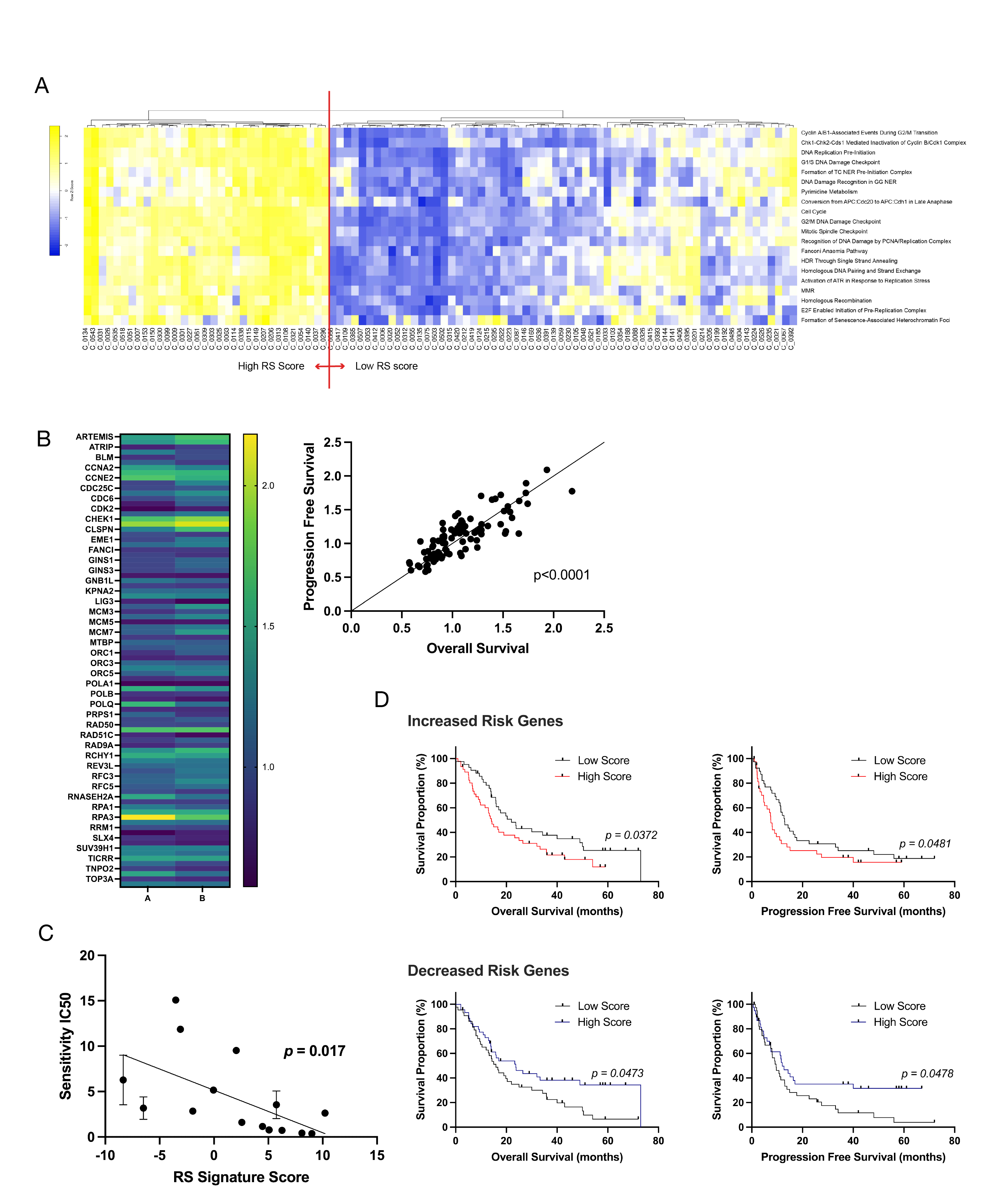

Supplement: Supplementary file 3 — Supplementary Figure S1 [file 41416_2025_3051_MOESM3_ESM.jpg]

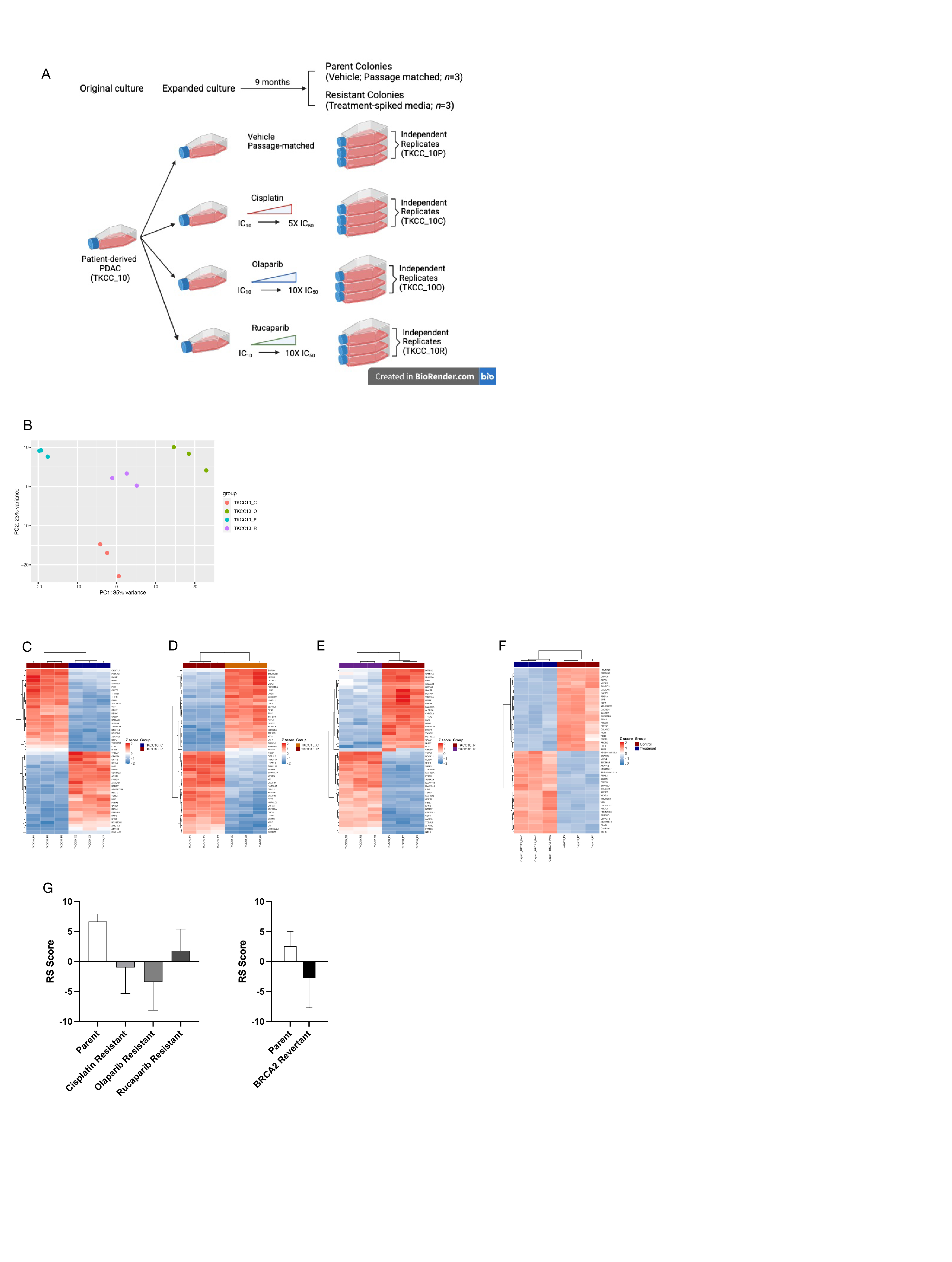

Supplement: Supplementary file 4 — Supplementary Figure S2 [file 41416_2025_3051_MOESM4_ESM.jpg]

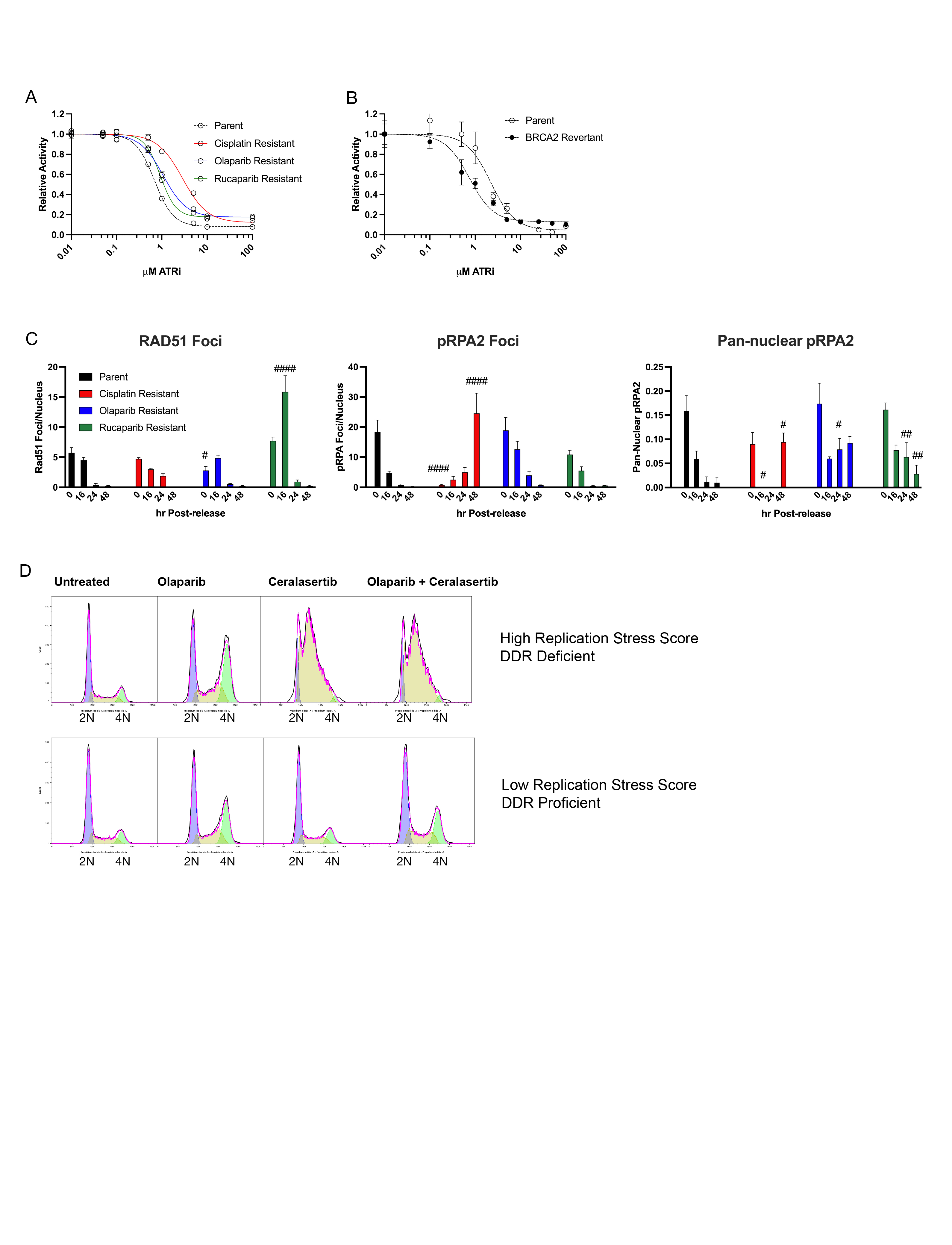

Supplement: Supplementary file 5 — Supplementary Figure S3 [file 41416_2025_3051_MOESM5_ESM.jpg]

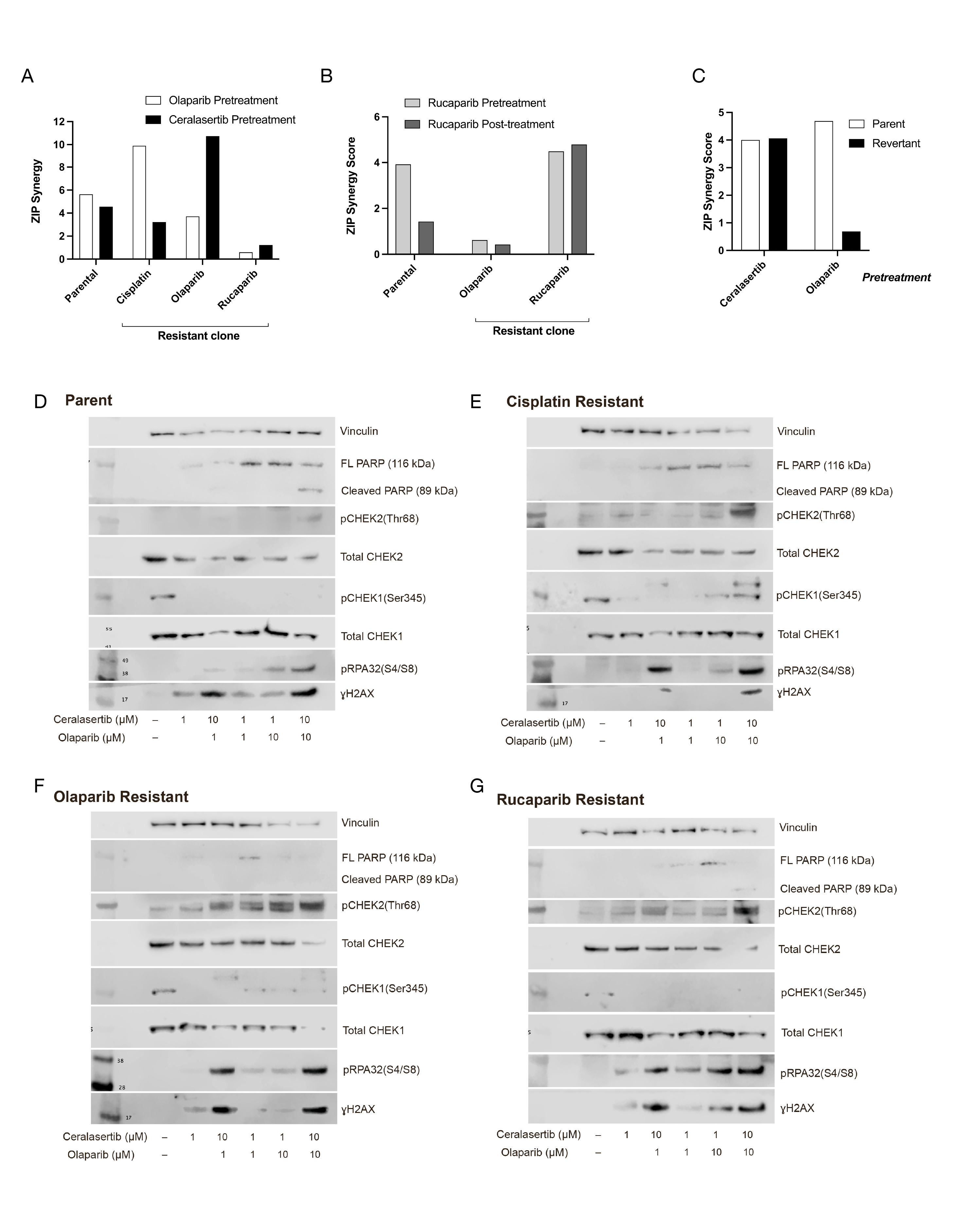

Supplement: Supplementary file 6 — Supplementary Figure S4 [file 41416_2025_3051_MOESM6_ESM.jpg]

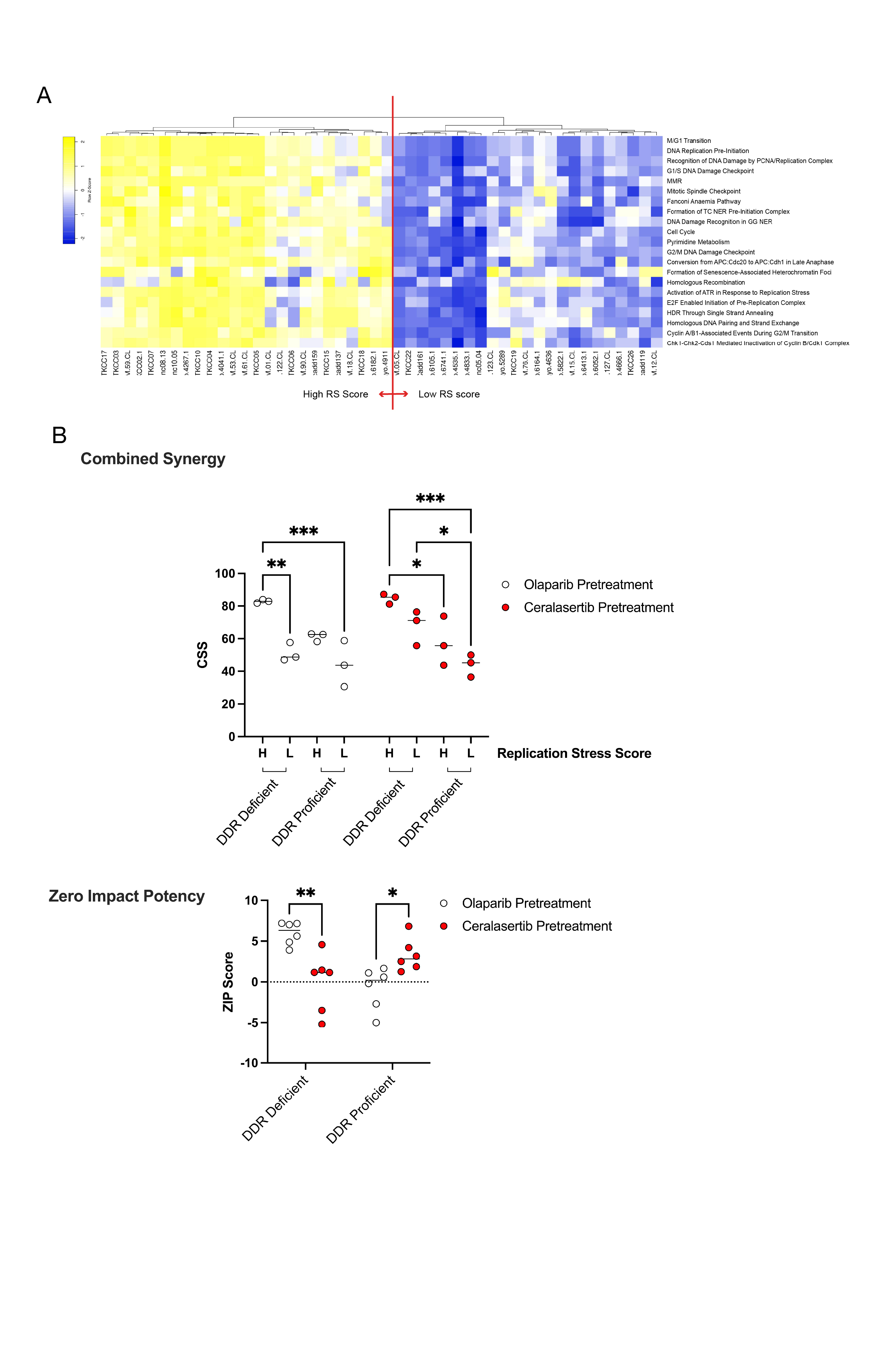

Supplement: Supplementary file 7 — Supplementary Figure S5 [file 41416_2025_3051_MOESM7_ESM.jpg]
